# Combination of Corn Pith Fiber and Biobased Flame Retardant: A Novel Method toward Flame Retardancy, Thermal Stability, and Mechanical Properties of Polylactide

**DOI:** 10.3390/polym13101562

**Published:** 2021-05-13

**Authors:** Yunxian Yang, De-Yi Wang, Laia Haurie, Zhiqi Liu, Lu Zhang

**Affiliations:** 1 Beijing Institute of Technology Chongqing Innovation Center, Chongqing 401120, China; 2 State Key Laboratory of Explosion Science and Technology, Beijing Institute of Technology, Beijing 100081, China; 3 Barcelona School of Building Construction (EPSEB), Universitat Politècnica de Catalunya, Av. Doctor Marañon 44, 08028 Barcelona, Spain; laia.haurie@upc.edu; 4 IMDEA Materials Institute, C/Eric Kandel, 2, 28906 Getafe, Madrid, Spain; lkeyi@126.com (Z.L.); zhangl@csu.edu.cn (L.Z.)

**Keywords:** biobased material, corn pith fiber, polylactide, flame retardancy, thermal stability

## Abstract

Some crop by-products are considered to be promising materials for the development of novel biobased products for industrial applications. The flammability of these alternatives to conventional materials is a constraint to expanded applications. Polylactide (PLA) composites containing a combination of oxidized corn pith fiber (OCC) and a biobased flame retardant (PA-THAM) have been prepared via an in situ modification method. SEM/EDS, FTIR and TGA were performed to establish that PA-THAM was coated onto the surface of OCC. The mechanical properties, thermal stability and fire behavior of PLA-based biocomposites were investigated. The incorporation of 5 phr PA-THAM imparted biocomposite good interfacial adhesion and increased decomposition temperature at 10% mass loss by 50 °C. The flame retardant properties were also improved, as reflected by an increased LOI value, a UL-94 V-2 rating, reduction of PHRR, and increased formation of char residue. Therefore, the introduction of 5 phr PA-THAM can maintain a good balance between flame retardancy and mechanical properties of this PLA/OCC system.

## 1. Introduction

Currently, environmental and sustainable awareness is stimulating the development of novel eco-friendly materials, which possess acceptable properties, biodegradability, low density and low cost [1]. Among these alternatives, natural fiber reinforced plastic composites (NPCs) are finding increasing acceptance and demand for structural applications [2,3,4].

The matrix plays an important role in a fiber-reinforced composite, most of which are thermoplastics. Among biobased materials, polylactide (PLA) is one of the main thermoplastics for the preparation of NPCs, and many efforts have been undertaken to investigate the preparation and properties of biocomposite for this matrix [5,6]. The introduction of two kinds of fiber into PLA generates a material that exhibits tensile specific strength, and stiffness increased by 116% and 62%, respectively, in comparison with the same properties for unreinforced PLA [7]. As a nucleating agent [8], the presence of natural cellulosic fiber enhances the crystallinity and melt strength of PLA-based biocomposites. A natural fiber/PLA system containing a phosphorus flame retardant, which has a metal–organic framework (MOF) structure, shows a 43% reduction in smoke production after combustion and improvement in tensile and impact strength compared to the same properties for pure PLA. A 5 wt % loading of flame retardant was required [9].

Recently, some crop by-products have been utilized in the furniture and building fields [10,11]. Corn, as a dominant industrial crop, can yield abundant corn stalk residue after harvest. Burning of this residue is still a common practice. Recycling of this by-product is an effective way to solve both an environmental and energy problem. A new biocomposite based on magnesium phosphate cement and corn stalk fiber has been developed for applications in insulation and structural units [12], which exhibits a better thermal and mechanical properties than the comparable material from hemp and cement. As a raw material [13], corn stalk residue was processed to extract a xerogel, which was incorporated into an epoxy system. This epoxy composition exhibits a high thermal stability, and upon decomposition yields significant char residue at 600 °C. The durability properties of concretes containing corn stalk residue have been studied [14]. It was determined that the utilization of biobased fillers can improve many engineering properties of concrete.

In addition, as a promising new generation material, the flammability of NPCs must be addressed. To enhance the fire resistance of NPC, the introduction of flame retardants is considered as an efficient and simple method [15]. Many phosphorus- and nitrogen-containing flame retardants can dramatically improve the fire resistance of polymeric materials due to their cooperative effects [16,17]. A novel eco-friendly flame retardant (PA-THAM) containing phosphorus and nitrogen has been synthesized [18]. The effect of the presence of PA-THAM on the properties of PLA has been assessed. Incorporation of only 3 wt % loading of this flame retardant produces a PLA biocomposite with excellent fire resistance and significant lubrication, with little change of mechanical properties. As the affinity between filler and matrix is also a crucial factor for the properties of a multi-phase system, PA-THAM was proposed to improve both the interfacial compatibility and flame retardant properties of the biocomposite.

An efficient in situ modification of natural fiber from corn stalk to incorporate novel eco-friendly flame retardant has been developed. The fiber structure after modification was characterized. This modified fiber was introduced into a PLA matrix. The mechanical properties, thermal stability, and fire behavior of the reinforced PLA were also investigated. Moreover, the relevant values of pure PLA, which was obtained previously under the same experiment conditions [18], are also listed to assess the effect of the combination of OCC and PA-THAM.

## 2. Materials and Methods

### 2.1. Materials

Polylactide (PLA, 4043D), extrusion grade, was supplied by NatureWorks (Minnetonka, MN, USA). NaOH (pellets, 98%) was purchased from Alfa Aesar (Waltham, MA, USA). Biobased flame retardant (PA-THAM), which was previously synthesized from Trometamol (THAM) and phytic acid (PA), was used directly in this study. The corn stalk residue was collected from a field in Castelló d’Empúries in the north of Catalonia.

### 2.2. Preparation of Oxidized Corn Pith Fiber (OCC)

Corn pith fiber (CC) was extracted from the soft, spongy tissue, which is at the interior of the corn stalk and has a noticeably low density. In order to improve the reactivity and thermal stability, this fiber was pre-treated by physical and chemical methods [19]. CC of millimeter size was obtained by mechanical milling; then oxidization was carried out in NaOH aqueous solution (PH = 11) at room temperature for 12 h with continuous stirring [20]. Finally, OCC was collected by filtration and washed with deionized water until neutral.

### 2.3. Modification of OCC with Flame Retardant

**Figure 1:**
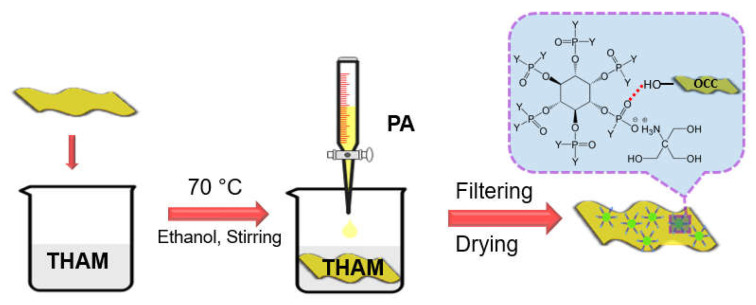
Scheme diagram of OCC modification with PA-THAM.

As shown in Figure 1, OCC was modified via in situ reaction of PA and THAM in suspension. THAM was firstly dissolved in ethanol at 70 °C; then added OCC and stirred to achieve a good dispersion. A corresponding equivalent of PA was dropped into the solution. The modified material was collected by filtration and washed with ethanol to remove the residual reagents; finally, OCC modified with different content of PA-THAM was obtained after drying at 80 °C and reduced pressure.

### 2.4. Preparation of Natural Fiber Reinforced Plastic Composites (NPCs)

Based on Table 1, PLA-based NPCs were fabricated via melt mixing method on a micro-compounder machine (MC 15, Xplore) at 170 °C for 3 min with a speed of 80 rpm. All the testing specimens were hot-pressed at 170 °C for 5 min into required dimensions according to correlative measurements. All involved materials were dried in a reduced pressure atmosphere at 80 °C for 8 h before use.

### 2.5. Characterization

Scanning electron microscopy coupled with energy dispersive spectrometer (SEM/EDS) was carried out to investigate the morphology and elements of OCC and NPCs on an apparatus (Helios NanoLab 600i, FEI: Waltham, MA, USA). The surface of both fiber and impact fracture of biocomposites was coated with 15 nm gold layer before observation at 10 kV accelerating voltage.

Fourier transforms infrared spectrometry (FTIR, Nicolet iS50: Waltham, MA, USA) technique was used to characterize the structure of OCC. The spectrum was recorded by scanning a range from 4000 cm^−1^ to 500 cm^−1^ on KBr powder. Thermogravimetric analysis (TGA) was conducted on an equipment (Q50, TA Instruments: New Castle, DE, USA) under a nitrogen flow of 90 mL/min. Samples with 10 ± 0.5 mg were placed in the furnace with a heating rate of 10 °C/min from room temperature to 700 °C. Furthermore, TGA connected with FTIR (TGA/FTIR) was also driven to investigate the pyrolyzed volatiles during thermal degradation, and all the experimental conditions were set as the same as previous FTIR and TGA tests.

Tensile test of PLA-based NPCs was performed by using the instruments INSTRON 3384 USA (Norwood, MA, USA) according to ASTM D 638-2014 with a crosshead speed of 5 mm/min at room temperature. Meanwhile, un-notched Charpy impact test was carried out on an impact equipment CEAST-Germany according to ISO 179-1-2010. For each formulation, at least 5 specimens were prepared and the average values were reported.

Dynamic mechanical analysis (DMA) was used to study the thermo-dynamic behavior on an instrument (TA Q800, single-cantilever mode). The testing conditions were set at the temperature from −40–120 °C with a heating rate 2 °C/min in frequency 1 Hz.

The limiting oxygen index (LOI), UL-94 vertical burning (UL-94) and cone calorimeter test (CCT) were carried out to characterize the fire performance of PLA-based biocomposites. LOI test was performed for specimens with the dimensions of 127.0 mm × 6.5 mm × 3.0 mm on an apparatus (FTT, UK: West Sussex, RH19 2HL, UK) according to ASTM D 2863-2013. UL-94 was conducted for the plate sheets with 127.0 mm × 13.0 mm × 3.0 mm on a fire instrument (FTT, UK) in accordance with ASTM D 3801-2010 procedure. CCT was used for samples (100.0 mm × 100.0 mm × 3.0 mm) on the basis of ISO 5660-1-2016 with a heat flux of 35 kW/m^2^.

## 3. Results and discussion

### 3.1. Analysis of Chemical Composition for Untreated and Treated OCC

**Figure 2:**
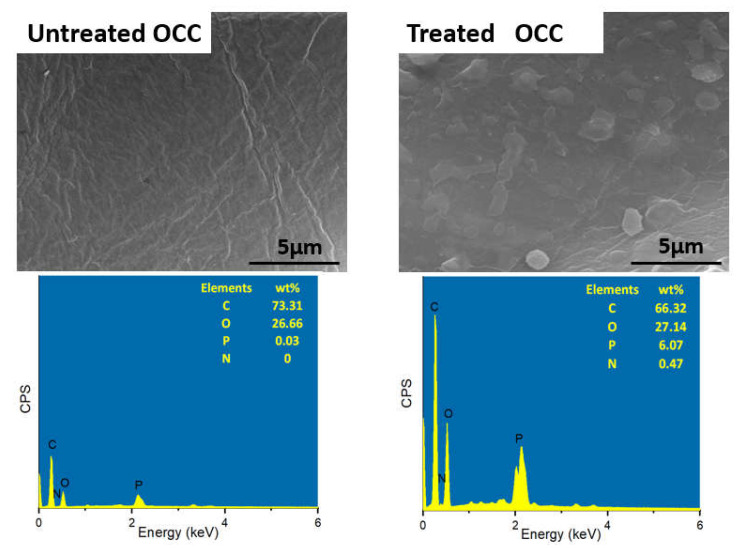
Images from SEM/EDS for the surface of OCC before and after modification.

Images from Figure 2 illustrates the microstructure of untreated and treated OCC. There was obvious difference between untreated and treated fiber surface. OCC exhibited a smooth surface with 73.31 wt % of carbon, 26.66 wt % of oxygen and little phosphorus which might result from the original composition in the corn pith. After modification, some small particles were coated on the surface of natural fiber, and additional elements comprised 6.07 wt % of phosphorus and 0.47 wt % of nitrogen were detected except carbon and oxygen.

**Figure 3:**
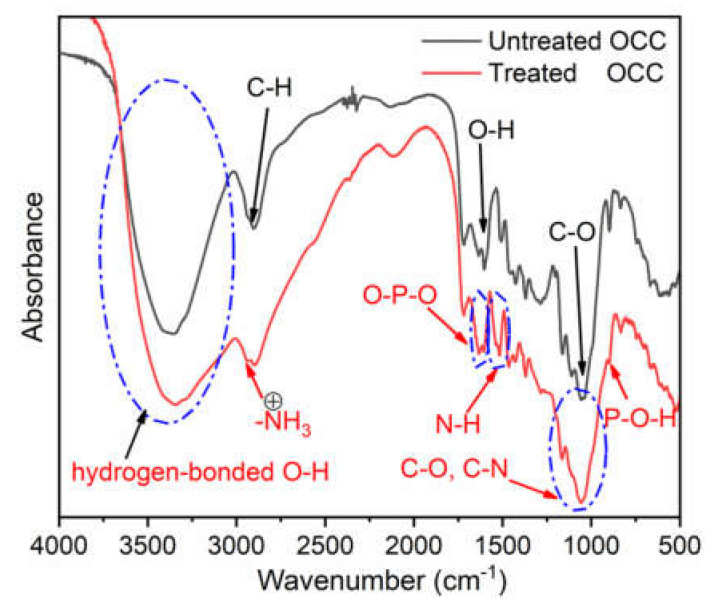
Infrared spectra of FTIR for untreated and treated OCC.

In order to further confirm the micro-structure of fiber surface, FTIR was utilized to determine the relative absorption peaks of functional groups. In comparison with the untreated OCC, some obvious changes can be observed in the curve of modified OCC except the typical absorption spectrum from natural fiber [21]. As shown in Figure 3, a wider and stronger absorbance from 4000 cm^−1^ to 3000 cm^−1^ is due to the formation of more hydrogen-bonded bands between PA-THAM and OCC. Bands at 2940 cm^−1^ caused by the stretching vibration of -NH_3_ in ammonium salts and 1530 cm^−1^ due to bending vibration of N-H were also detected [22]. Meanwhile, other obvious absorption peaks from stretching vibration of O-P-O and stretching vibration of P-O-H were observed at 1630 cm^−1^ and 890 cm^−1^, respectively [23]. Besides, the overlap from bending modes of C-O and C-N bonds leads to another wide and strong characteristic band occurred around 1060 cm^−1^, which shifted to higher wavenumber owing to the formation of hydrogen-bonded bands [23,24]. The appearance of these additional elements and characteristic peaks proves that the PA-THAM was coated onto the surface of OCC.

### 3.2. Thermal Degradation Behavior of Untreated and Treated OCC

**Figure 4:**
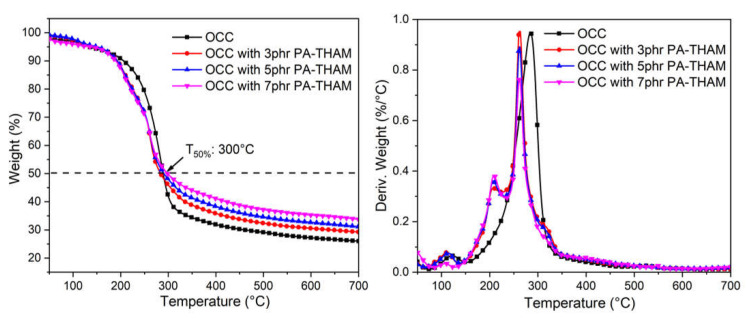
Results from thermogravimetric test for OCC before and after modification.

The thermal behavior of OCC before and after modification was studied by thermogravimetric analysis, shown in Table 2 and Figure 4. Both untreated and treated OCC comprised three stages during degradation process [25]. The evaporation of free water occurs at around 100 °C. Secondary stage between 200 °C and 350 °C is caused by hemicellulose and cellulose decomposition; then the last thermal event is attributed to the degradation of lignin, which exhibited a small and slow mass loss at a wide range of temperature [26]. After modification with PA-THAM, the temperature corresponding to the decomposition of hemicellulose and cellulose moved to a lower value, which can be attributed to the premature decomposition of these two contents caused by PA-THAM. However, the maximum degradation rate was reduced, and more than 30 wt % residue was left after 300 °C. This is because PA-THAM releases phosphoric acid products at lower temperature to induce carbonization of the corn pith fiber by dehydration reaction; then the formation of char layer protects the inner materials from further decomposition [27,28]. On account of thermal behavior, the modified corn pith fiber can be used as the filler for PLA.

### 3.3. Mechanical Properties

The variation of tensile and impact properties for PLA and PLA-based NPCs is listed in Table 3. Generally, the introduction of corn pith fiber into PLA decreased the mechanical properties of NPCs except Young’s Modulus. This is attributed to the incompatibility between fiber and matrix. Compared with NPC0, tensile properties of sample with 5 phr content of PA-THAM was hardly affected, which varied from 3.1% to 3.0% for elongation at break and from 54.1 MPa to 53.7 MPa for tensile strength, as well as had same value for Young’s modulus. This can be explained that the small loading of flame retardant had little effect on the crystallinity of biocomposites.

**Figure 5:**
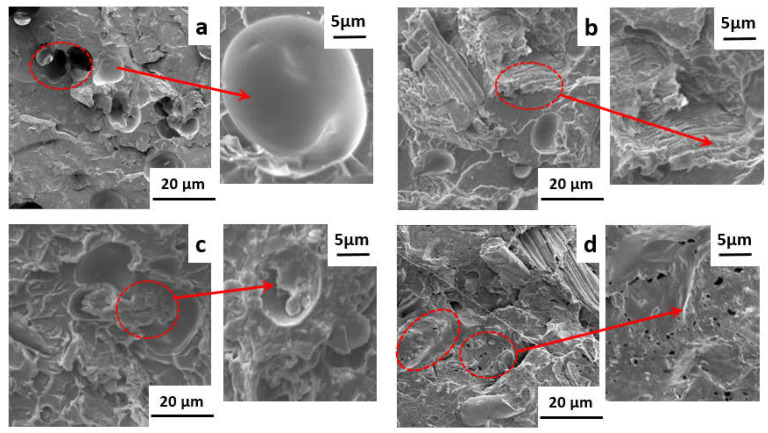
SEM morphology of impact-fracture surface for PLA-based NPCs: (**a**) NPC0; (**b**) NPC1; (**c**) NPC2; (**d**) NPC3.

However, an initial upward and following downward trend was illustrated by impact strength data of NPCs. The sample NPC0 exhibited a value of 11.7 kJ/m^2^. After incorporating modified OCC into PLA, this performance was enhanced by a higher value of 13.0 kJ/m^2^ at 5 phr loading of PA-THAM. While the further addition of flame retardant resulted in a reduction, which presented 11.8 kJ/m^2^ for NPC3 with 7 phr PA-THAM. In order to elucidate this phenomenon, SEM was performed to investigate the micro-morphology of the biocomposites (shown in Figure 5), which is of importance to investigate the properties for multiphase material system.

According to Figure 5a, NPC0 with only PLA and OCC demonstrated a poor compatibility with many smooth holes and clean fiber surface. As a result, these defects in weak region is prone to lead a prior failure and debonding. After introducing the modified OCC into PLA, Figure 5b,c illustrated that the debonding holes decreased and were replaced by fibers adhered with PLA resin; meanwhile, the continuous matrix-phase also changed from a flat surface to a rough one. Additionally, the fiber was embedded well in the matrix with an obscured boundary, and homogeneous morphology with little aggregation was achieved. These phenomena indicates that the incorporation of PA-THAM increased the interfacial affinity and resulted in a good dispersion simultaneously [29,30,31]. As a compatibilizer, when PA-THAM was further added into the PLA-based biocomposite (NPC3), some big agglomerates and discontinuous droplets were observed in Figure 5d. This is probably because when the ratio between OCC and PA-THAM exceeds some range, the interfacial interaction is dominated by PLA and PA-THAM in this ternary system [32,33]. Thus, an optimum addition of 5 phr PA-THAM can maintain the mechanical properties of biocomposites.

**Figure 6:**
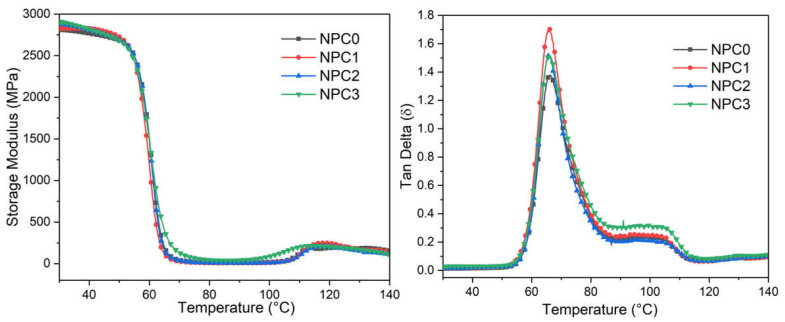
Results from DMA test for PLA-based NPCs.

From the curves in Figure 6, all the PLA-based biocomposites illustrated two similar thermo-dynamic relaxation processes. The first peak around 60 °C is due to the glass-to-rubber transition, and the other one at approximate 110 °C is attributed to secondary transition behavior derived from the crystal phase. With respect to the storage modulus of PLA-based NPCs, all the samples presented a constant value in the glassy region. When the temperature rose up to 60 °C, the value declined significantly caused by glass-transition, and then increased a little at around 110 °C corresponding to the cold crystallization [34].

However, the curves from Tan Delta, which is correlated with polymer chains relaxation, demonstrated a higher value of tan δ peak height with the content of PA-THAM. This is because the incorporation of PA-THAM introduces the polar groups, and more time is needed to overcome the friction between the molecular chains [35]. When the addition of PA-THAM continuously increased, the peak intensity of tan δ fell down, which is because the lubrication effect from PA-THAM weakens the intermolecular interaction [36,37]. Moreover, the peak width at half height of the biocomposites, which is considered as a criterion for hinting interaction between components in the multiphase system, increased with the content of PA-THAM [37]. This can be also clarified with a good interaction between PLA and fillers, as was analyzed in the previous part [38].

### 3.4. Thermal Stability

**Figure 7:**
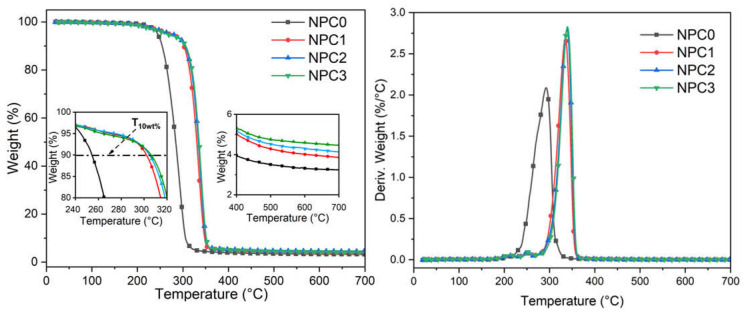
Results from thermal gravimetric analysis for PLA-based NPCs.

An obvious difference in thermal stability can be seen in Figure 7 before and after incorporation of PA-THAM. Compared with pure PLA (T_10%_: 330 °C), a premature decomposition of NPC0 occurred at a lower temperature (T_10%_) of 255 °C due to poor thermal stability of fiber. While the decomposition temperature at 10% mass loss of PLA-based biocomposites increased significantly by 50 °C after adding the fiber modified with PA-THAM. This is owing to the phosphorylation of corn pith fiber by reacting with PA-THAM rather than premature thermal degradation induced by corn pith fiber [39,40]. Additionally, a protective char layer forms to restrict the penetration of volatiles out from the material at the early stage and lessen further thermal degradation [41].

However, due to the continuous heating, the protective layer starts to break down, which results in further decomposition of PLA and fillers; then a higher value of maximum decomposition rate in biocomposites with PA-THAM was observed than that in NPC0 [42]. When the temperature was up to 400 °C, the amount of char residue in the biocomposite with flame retardant was also more than that in the sample NPC0.

**Figure 8:**
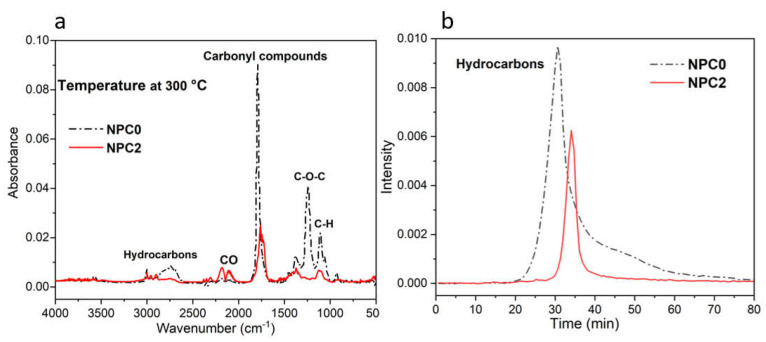
Curves from TGA/FTIR test for NPC0 and NPC2: (**a**) FTIR spectrum for volatiles at 300 °C; (**b**) evolution of hydrocarbons component at the whole heating process.

In order to analyze the significant improvement in thermal stability, TGA/FTIR was carried out to investigate the evolution of volatile products during thermal degradation process of PLA-based biocomposites. From Figure 8a, it can be seen that the incorporation of PA-THAM did not change the main thermal decomposed products [43], but the intensity of volatiles from NPC2 at 300 °C was much weaker that that from NPC0. Furthermore, Figure 8b shows the curves of intensity versus time during the whole heating process, which represents the evolution of relative concentration of hydrocarbon products [44]. It is evident that NPC0 released higher amounts of volatiles at earlier stages than NPC2 did. These data elucidate that the introduction of PA-THAM significantly postpones the degradation evolution of biocomposite and leads to an incomplete degradation, which is consistent with the previous results.

On the base of the analysis for thermal performance, it is indicated that the combination of OCC and PA-THAM can improve the thermal stability by forming a protective layer and prevent a premature degradation of biocomposites [45,46].

### 3.5. Fire Behavior

The combustion behaviors were firstly evaluated by LOI and UL-94 tests, and the results are presented in Table 4.

In comparison with pure PLA, almost no enhancement was observed in sample NPC0, showing a LOI value of 19.0 and burning out in UL-94 with no rating. However, it is obvious that the presence of PA-THAM improved the LOI value as well as the rating in UL-94 test. With the increasing concentration of PA-THAM, PLA-based biocomposites illustrated an upward trend of LOI value and achieved V-2 rating in UL-94 test for NPC2 and NPC3. This is attributed to the cooperative effect between corn pith fiber and flame retardant. Although the addition of 7 phr PA-THAM reduced the total flaming time, the result in UL-94 test only conserved V-2 rating. This is due to the occurrence of flaming dripping during combustion, which is caused by the extractable substance from corn pith fiber [47].

**Figure 9:**
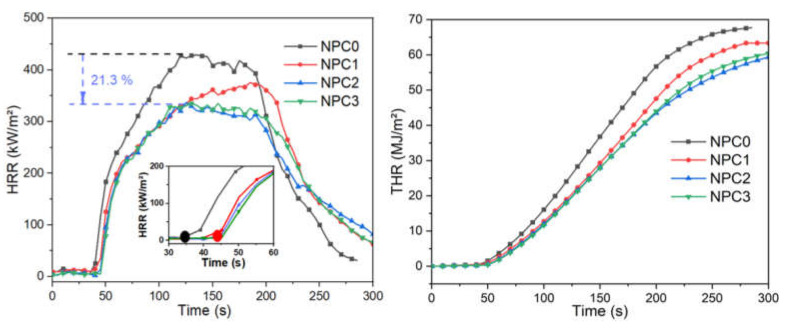
Curves of HRR and THR vs. time from cone calorimeter test for PLA-based NPCs.

As for cone calorimeter test, which well represents a developing fire scenario and is widely used for researching the fire behavior of polymer materials [47,48], some important parameters are summarized in Table 5 and Figure 9, such as heat release rate (HRR), peak heat release rate (PHRR), time to ignition (TTI), total heat release (THR), average effective heat of combustion (Av-EHC) and residue after combustion.

Sample NPC0 without PA-THAM exhibited a flammable behavior with starting to ignition at 35 s, combusting completely, releasing THR of 65 MJ/m^2^ with a PHRR of 428 kW/m^2^ and only 0.5 wt % residue, which is even worse than the data from pristine PLA [18,49]. This phenomenon results from the poor thermal stability of the fiber. Whereas the addition of PA-THAM generally improved the flame retardant properties of PLA-based biocomposites, especially for NPC2 with 5phr PA-THAM. Compared with NPC0, the value of PHRR reduced by 21.3%, 6.2 wt % char residue left, and TTI was delayed by 8 s.

Ignition of polymer materials, as thermally thin PLA-based samples, can be analyzed by physical and chemical characterization according to Equation (1) [50]. In this case, the physical factors (ιρc) can be considered constant due to the same dimensions and small loading of flame retardant. Nevertheless, on account of the same experimental conditions, the TTI value of all the biocomposites with PA-THAM was higher than that of the sample NPC0. Therefore, the chemical factor CHF, which corresponds to thermal stability, would affect the final TTI value. This can be elucidated that the incorporation of PA-THAM improves the thermal stability of biocomposite by forming carbonaceous layer; and then prevents the premature decomposition of corn pith fiber as well as decreases the concentration of volatiles [51], which is also consistent with the results from TGA.

(1) $$\mathrm{TTI}=\iota \rho c\frac{T_{ig}-T_{0}}{q{\prime \prime }_{ext}-CHF}$$

where ι is the sample thickness; ρ is the density; c is the heat capacity; $T_{ig}$ and $T_{0}$ are the ignition temperature and initial ambient temperature, respectively; $q’{’}_{ext}$ is the experimental heat flux, while CHF is the critical heat flux.

**Figure 10:**
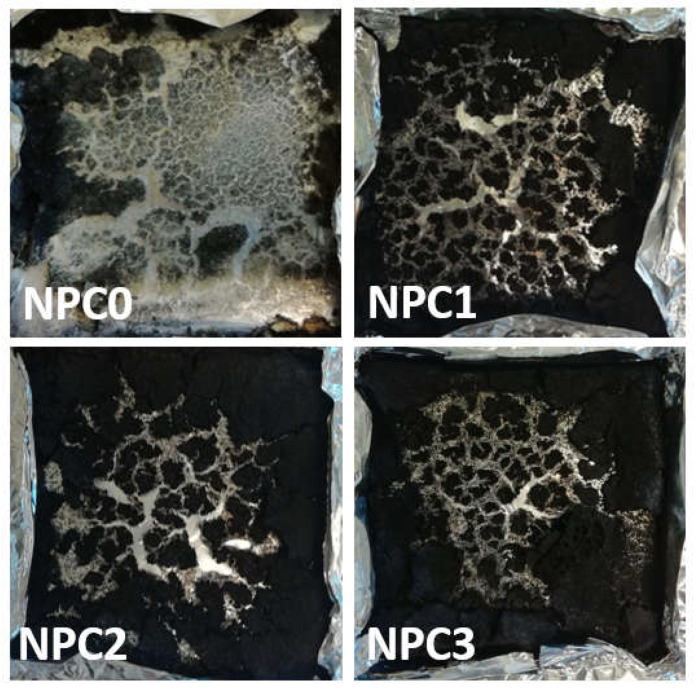
Digital photos of char residue for PLA-based NPCs after cone calorimeter test.

**Figure 11:**
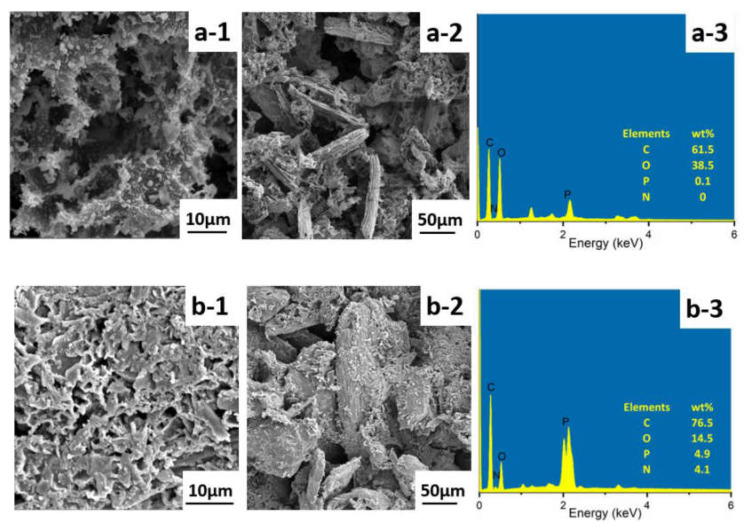
Morphology and elements analysis of the char residue surface after cone calorimeter test for NPC0 and NPC2: (**a-1**) external part of NPC0; (**a-2**) internal part of NPC0; (**a-3**) EDS for NPC0 char residue; (**b-1**) external part of NPC2; (**b-2**) internal part of NPC2; (**b-3**) EDS for NPC2 char residue.

Furthermore, the value of Av-EHC, which indicates the amounts of released volatiles during combustion, presented little change for all the samples. Consequently, the main flame retardant mechanism can be determined by investigating char residue after cone calorimeter test [52]. Figure 10 and Figure 11 gave the digital photos for PLA-based NPCs and micro-morphology images for NPC0 and NPC2 after combustion, respectively. There was a little char residue for NPC0, while higher amounts left for other biocomposites with modified OCC and the value increased with the content of PA-THAM.

The residue of NPC0 demonstrated a discontinuous and loose surface with big voids. By comparison, both external and internal surface of NPC2 presented a more continuous and compact morphology structure. Additionally, more phosphorus and nitrogen elements were detected in char residue of NPC2. Therefore, the investigation of char layer elucidates that the combination of OCC and PA-THAM enables NPC2 to possess not only good dispersion, but also better flame retardant properties, which is because of a cooperative effect in the condensed phase [53,54].

## 4. Conclusions

A biobased flame retardant (PA-THAM) was successfully coated onto the surface of oxidized corn pith fiber (OCC) via in situ modification. This modified OCC was introduced to PLA to prepare natural fiber reinforced plastic composites (NPCs). Afterwards, the effect of the combination of PA-THAM and OCC on the NPCs’ properties was investigated systematically. In comparison with the sample NPC0, the biocomposite with 5 phr loading of PA-THAM (NPC2) showed a 50 °C higher value of the decomposition temperature at 10% mass loss, and exhibited better impact strength. Moreover, the LOI value increased from 19.0% to 25.2% and the classification in UL-94 varied from no rating to V-2 rating. A 21.3% reduction of PHRR, increased char residue and delayed TTI were also observed in CCT. When the content of PA-THAM rose up to 7 phr, the thermal stability and flame retardancy of biocomposite (NPC3) were enhanced a little, while the mechanical properties decreased obviously. In conclusion, to some extent, the presence of PA-THAM contributed to an improvement of the NPC’s properties, and the 5 phr loading of PA-THAM can be an optimum formulation for this NPC system to achieve the balance of flame retardancy, thermal stability and mechanical properties.

Regarding to the PLA-based composite, although the combination of OCC and PA-THAM did not offset the deterioration of the material’s performance caused by corn pith fiber, the compatibility of corn pith fiber and PLA and flame retardancy were improved indeed. Therefore, optimizing the original performance of corn pith fiber, such as particle size, could be the following research interest to further enhance the comprehensive properties of this NPC composite.

## Figures and Tables

**Table 1:** Formulations for PLA-based NPCs.

Sample	PLA/(wt %)	OCC/(wt%)	PA-THAM */(phr)
NPC0	90	10	/
NPC1	90	10	3
NPC2	90	10	5
NPC3	90	10	7

* PA-THAM was fixed with phr on the basis of total composite mass.

**Table 2:** Results from TGA of OCC with and without FR (PA-THAM).

Sample	T1 */(°C)	T2 */(°C)	Residue (wt %) at	
			400 °C	700 °C
OCC	120	240–282	30.5	25.5
OCC with 3phr FR	110	209–262	35.9	29.4
OCC with 5phr FR	109	210–262	38.5	31.1
OCC with 7phr FR	109	209–261	40.8	34.1

T1 *: the temperature at free water’s evaporation. T2 *: the temperature at hemicellulose and cellulose’s decomposition.

**Table 3:** Mechanical properties of PLA-OCC NPCs.

Sample	Elongation at Break/(%)	Tensile Strength/(MPa)	Young’s Modulus/(GPa)	Impact Strength/(kJ/m^2^)
PLA *	4.1 ± 0.1	67.3 ± 1.5	2.2 ± 0.2	23.6 ± 2.0
NPC0	3.1 ± 0.1	54.1 ± 2.0	2.4 ± 0.1	11.7 ± 1.0
NPC1	2.9 ± 0.1	52.4 ± 1.0	2.4 ± 0.2	12.1 ± 0.5
NPC2	3.0 ± 0.1	53.7 ± 1.0	2.4 ± 0.1	13.0 ± 0.2
NPC3	2.1 ± 0.2	45.7 ± 2.0	2.3 ± 0.1	11.8 ± 0.2

* The values of pure PLA are from the reference [18]. Copyright 2020. Reproduced with permission from Express Polymer Letters.

**Table 4:** Results from LOI and UL-94 tests for PLA-OCC biocomposites.

Sample	LOI/(%)	UL-94			
		$\bar{t1+t2}/(s)$	Rate	Ignition	Dripping
PLA *	19.9±0.2	Burt out	NR *	Yes	Yes
NPC0	19.0±0.2	Burt out	NR	Yes	Yes
NPC1	22.0±0.2	10 + 60	NR	Yes	Yes
NPC2	25.2±0.1	13 + 14	V-2	Yes	Yes
NPC3	26.1±0.1	14 + 0	V-2	Yes	Yes

* NR: NO Rating. * The values of pure PLA are from the reference [18]. Copyright 2020. Reproduced with permission from Express Polymer Letters.

**Table 5:** Results from CCT for PLA-OCC biocomposites.

Sample	PHRR/(kW/m²)	TTI/(s)	THR/(MJ/m^2^)	Av-EHC/(MJ/kg)	Residue/(wt%)
PLA *	410 ± 5	61 ± 1	70 ± 1	15.7 ± 0.1	0.6
NPC0	428 ± 6	35 ± 2	65 ± 2	16.5 ± 0.1	0.5
NPC1	375 ± 5	43 ± 2	62 ± 1	16.6 ± 0.1	3.9
NPC2	337 ± 3	43 ± 2	58 ± 1	16.4 ± 0.1	6.2
NPC3	335 ± 4	44 ± 2	57 ± 2	16.1 ± 0.2	6.4

* The values of pure PLA are from the reference [18]. Copyright 2020. Reproduced with permission from Express Polymer Letters.
